# Comprehensive review of the evidence regarding the effectiveness of community–based primary health care in improving maternal, neonatal and child health: 8. summary and recommendations of the Expert Panel

**DOI:** 10.7189/jogh.07.010908

**Published:** 2017-06

**Authors:** Robert E Black, Carl E Taylor, Shobha Arole, Abhay Bang, Zulfiqar A Bhutta, A Mushtaque R Chowdhury, Betty R Kirkwood, Nazo Kureshy, Claudio F Lanata, James F Phillips, Mary Taylor, Cesar G Victora, Zonghan Zhu, Henry B Perry

**Affiliations:** 1 Johns Hopkins Bloomberg School of Public Health, Baltimore, Maryland, USA; 2 Jamkhed Comprehensive Rural Health Project, Jamkhed, Maharashtra, India; 3 Society for Education, Action and Research in Community Health, Gadchiroli, Maharashtra, India; 4 University of Toronto, Toronto, Ontario, Canada; 5 Aga Khan University, Karachi, Pakistan; 6 BRAC, Dhaka, Bangladesh; 7 London School of Hygiene and Tropical Medicine, London, United Kingdom; 8 Bureau of Global Health, United States Agency for International Development, Washington, DC, USA; 9 Institute of Nutritional Research, Lima, Peru; 10 Columbia University Mailman School of Public Health, New York, New York, USA; 11 Independent Consultant, South Royalton, Vermont, USA; 12 Federal University of Pelotas, Pelotas, Brazil; 13 Capital Institute of Pediatrics and China Advisory Center for Child Health, Beijing, China; * Chairperson, Expert Panel; † Former Chairperson, Expert Panel (deceased); ‡ Member, Expert Panel

## Abstract

**Background:**

The contributions that community–based primary health care (CBPHC) and engaging with communities as valued partners can make to the improvement of maternal, neonatal and child health (MNCH) is not widely appreciated. This unfortunate reality is one of the reasons why so few priority countries failed to achieve the health–related Millennium Development Goals by 2015. This article provides a summary of a series of articles about the effectiveness of CBPHC in improving MNCH and offers recommendations from an Expert Panel for strengthening CBPHC that were formulated in 2008 and have been updated on the basis of more recent evidence.

**Methods:**

An Expert Panel convened to guide the review of the effectiveness of community–based primary health care (CBPHC). The Expert Panel met in 2008 in New York City with senior UNICEF staff. In 2016, following the completion of the review, the Panel considered the review’s findings and made recommendations. The review consisted of an analysis of 661 unique reports, including 583 peer–reviewed journal articles, 12 books/monographs, 4 book chapters, and 72 reports from the gray literature. The analysis consisted of 700 assessments since 39 were analyzed twice (once for an assessment of improvements in neonatal and/or child health and once for an assessment in maternal health).

**Results:**

The Expert Panel recommends that CBPHC should be a priority for strengthening health systems, accelerating progress in achieving universal health coverage, and ending preventable child and maternal deaths. The Panel also recommends that expenditures for CBPHC be monitored against expenditures for primary health care facilities and hospitals and reflect the importance of CBPHC for averting mortality. Governments, government health programs, and NGOs should develop health systems that respect and value communities as full partners and work collaboratively with them in building and strengthening CBPHC programs – through engagement with planning, implementation (including the full use of community–level workers), and evaluation. CBPHC programs need to reach every community and household in order to achieve universal coverage of key evidence–based interventions that can be implemented in the community outside of health facilities and assure that those most in need are reached.

**Conclusions:**

Stronger CBPHC programs that foster community engagement/empowerment with the implementation of evidence–based interventions will be essential for achieving universal coverage of health services by 2030 (as called for by the Sustainable Development Goals recently adopted by the United Nations), ending preventable child and maternal deaths by 2030 (as called for by the World Health Organization, UNICEF, and many countries around the world), and eventually achieving Health for All as envisioned at the International Conference on Primary Health Care in 1978. Stronger CBPHC programs can also create entry points and synergies for expanding the coverage of family planning services as well as for accelerating progress in the detection and treatment of HIV/AIDS, tuberculosis, malaria, hypertension, and other chronic diseases. Continued strengthening of CBPHC programs based on rigorous ongoing operations research and evaluation will be required, and this evidence will be needed to guide national and international policies and programs.

This paper summarizes the current evidence regarding the effectiveness of community–based primary health care (CBPHC) in improving maternal, neonatal and child health (MNCH). It also proposes concrete steps to recognize that communities are a vital resource and key partners with health systems in improving MNCH.

We summarize here the findings presented in the earlier articles in this current series [1–7] and in the Reproductive, Maternal, Newborn and Child Health volume of the Disease Control Priorities, Third Edition [8,9]. It also is an outgrowth of the Working Group on CBPHC of the International Health Section of the American Public Health Association, and the papers of the Working Group prepared previously [10–12] as well as discussions of an Expert Panel (for membership see **Online Supplementary Document(Online Supplementary Document)**) convened to guide the activities of the Working Group when the Panel met at UNICEF headquarters in New York City on 27–8 March 2008 with senior UNICEF staff along with senior staff from the World Health Organization (WHO) and the World Bank.

CBPHC is defined as a process by which health systems work with communities to improve health through activities that may be linked with health facilities but which take place in communities. The role of communities and community–based approaches to improving MNCH is still being overshadowed by the traditional “facility–centric” approach to health systems and calls for a new paradigm in which communities and community–based services are brought to the mainstream of health programs in order to improve the effectiveness of health systems in resource–constrained settings. Hybrid approaches also need to be developed in which professionalized CHWs divide their time by attending to patients at a village–based health post and visiting families in their homes, as is the case in Ethiopia.

The previous articles in this series provide an in–depth comprehensive review of evidence accumulated for over half a century regarding the effectiveness of community–based primary health care (CBPHC) in improving maternal, neonatal and child health (MNCH). The authors identified assessments of the health effects of community–based projects, programs and research studies (hereafter referred to as projects) in defined geographic populations. The review defines health effects broadly: change in (1) the population coverage of evidence–based interventions, (2) nutritional status, (3) serious morbidity, and (4) mortality. Not only did the authors assess health effects, but they also examined the equity of these projects as well as the strategies used to achieve health effects, including the common strategies of four projects identified that had evidence of mortality impact for 10 years or longer.

## Why the review is important now

The era of the Millennium Development Goals (MDGs) ended in 2015 with only seven of 75 Countdown countries reaching the goal for maternal mortality and only one–third reaching the goal for mortality of children younger than five years of age [8]. The population coverage of 13 of 21 key evidence–based MNCH interventions is still less than 60% and for 6 of the 21 interventions it is less than 40% [8]. The second international *Countdown to 2015 Conference* met in Cape Town, South Africa, on 17–19 April 2008. The Call for Action arising from this Conference focused on the need for “long–term, predictable financing for strengthened health systems to deliver essential services to women, newborns and children,” “dramatic scale–up of high–impact interventions,” harmonization of donor support, and increased political commitment to health around the world [9]. However, there was no mention or call for building stronger partnerships with communities or strengthening CBPHC. Communities are the most undervalued resource in global health. Had communities been engaged more fully as partners with health systems, and had community–based primary health care been more fully developed, we believe there is a strong possibility that the MDG era might have ended very differently.

In 1948, the United Nations General Assembly affirmed in its Universal Declaration of Human Rights that everyone has a right to medical care and that “motherhood and childhood are entitled to special care and assistance” [13]. Forty years later, in 1978, the largest gathering of health officials convened up to that time by the World Health Organization and UNICEF affirmed at the International Conference on Primary Health Care that an acceptable level of health for all the people of the world could be achieved by the year 2000 through a fuller and better use of the world’s resources (see **Box 1**) [15]. As the world seeks still to achieve these lofty goals, much work remains to be done. In 2015 the United Nations has adopted the Sustainable Development Goals, calling for a world *“free of poverty, hunger, disease and want, where all lives can thrive”* by the year 2030, with universal access to “*quality essential health–care services”* [16]. The World Health Organization and UNICEF have called for ending preventable child and maternal deaths in a generation [17,18]. However, even though recently released plans for achieving this goal do emphasize the importance of community engagement/empowerment, the critical and fundamental contribution of CBPHC to achieving this goal is muted [19,20].

Box 1 **The Declaration of Alma Ata**

“The people have the right and duty to participate individually and collectively in the planning and implementation of their health care” (Article V).

“Primary health care is essential health care based on practical, scientifically sound and socially acceptable methods and technology made universally accessible to individuals and families in the community through their full participation and at a cost that the community and country can afford to maintain at every stage of their development in the spirit of self–reliance and self–determination” (Article VI).

Primary health care “requires and promotes maximum community and individual self–reliance and participation in the planning, organization, operation and control of primary health care, making fullest use of local, national and other available resources; and to this end develops through appropriate education the ability of communities to participate” (Article VII) [14].

## RESULTS

### Specific interventions

**Table 1** contains the evidence–based interventions that can be provided by community–level workers with appropriate training, supervision and support. All of these interventions are described in the review. The number of such interventions will certainly continue to grow with continued experience and operations research.

**Table 1:** Effective interventions for maternal, newborn and child health that can be provided by community health workers in the community or at a health post [21–23]

Point in continuum of care				
**Pregnancy**	**Delivery (normal)**	**Postpartum (woman)**	**Postpartum (newborn)**	**Child**
Preparation for safe birth and newborn care; emergency planning	Management of labor and delivery and referral of complications	Promotion of breastfeeding	Neonatal resuscitation	Promote breastfeeding and complementary feeding
Micronutrient supplementation*			Breastfeeding	Provide vitamin A, zinc, and food supplementation
Nutrition education			Thermal care for preterm newborns	Immunizations
Intermittent preventive treatment of malaria during pregnancy)			Promote care–seeking	Co–trimoxazole for HIV–positive children
Food supplementation			Assess for danger signs and refer	Education on safe disposal of children’s stools and handwashing
Promotion of HIV testing			Oral antibiotics for pneumonia	Distribute and promote use of ITNs† or IRS‡, or both
				Assess for danger signs and refer
				Detect and refer children with severe acute malnutrition
				Detect and treat serious infections without danger signs (iCCM§), refer if danger signs present

*Because of some evidence of risk and gaps in the evidence, the WHO does not at this time recommend multiple micronutrient supplementation for pregnant women to improve maternal and perinatal outcomes [24].

†Insecticide–treated bednet.

‡Indoor residual spraying.

§Integrated community case management (the components include treatments for diarrhea, pneumonia, malaria).

### Equity

Although the equity of CBPHC services have not been studied as extensively as has overall intervention effectiveness, the available evidence supports a strong pro–equity effect of CBPHC interventions, as described in more detail in Paper 5 of this series [5]. The term pro–equity effect signifies that the most disadvantaged segment of the population, usually defined in terms of income quintiles or some other type of socio–economic status, benefit more from the delivery of one or more CBPHC interventions than does the better–off segment of the population. Community–based approaches can reach those furthest from health facilities and can rapidly expand population coverage of key interventions, so these findings are not surprising. These findings stand in stark contrast to the commonly observed finding that utilization of primary health care facilities is inequitable because those in the lower income quintiles are less likely to obtain services there [25,26]. This evidence together with the lack of evidence that investments in facilities alone can improve population health in resource–constrained settings [27,28] provide additional support for the importance of investing in CBPHC for improving MNCH.

### Strategies for achieving effectiveness

The projects included in the review utilized myriad innovative approaches for working in partnership with communities and with health systems for making CBPHC interventions effective in improving MNCH. These are described in greater detail in paper 4 in this series [6]. Clearly no one size fits all situations, and contextual considerations have a major influence on project operations. Nevertheless, important themes emerged from the review. Many project assessments described engagement with community leaders (both formal and informal), engagement with existing and/or formation of new women’s groups, and devising innovative ways to share key education messages with the community (through skits, songs, stories, games and peer–to–peer education). Community–level workers of many types (including both volunteer and paid workers) assisted with project implementation. In most cases, these workers were women, and in three–quarters of the projects included in the review some type of training was provided to these workers. In more than half the projects assessed the community was involved in project implementation, promotion of partnerships between the project and the community, promotion of the use of local resources, and promotion of community engagement/empowerment. In nearly half of the projects, promotion of women’s empowerment was present. In approximately 39% of the projects, communities were involved in planning the project and in 40% they were involved in the evaluation.

Many projects engaged in health system strengthening activities of various types, including training of staff based at peripheral health facilities who supervise community–level activities and treat referred patients, strengthening the supervisory system of community–level workers and the logistics/drug supply system for both the peripheral health facility and the community–level workers, and strengthening the referral system. Building strong links among the community–level activities, the peripheral health facility and the referral hospital were common features of effective projects.

Finally, four implementation intervention strategies were commonly encountered. First, home visitations, often routine visits to all homes as well as visits to targeted groups, were often carried out by both volunteer and paid community–level workers. Second, these workers commonly provided community case management, in which they provided education on warning and dangers signs, identified cases in need of referral, and/or treated cases in the community with appropriate medications. A third strategy identified among the projects included in the assessments was the formation of participatory women’s groups in which groups of women meet with a facilitator to learn about ways in which they can promote their own health and the health of their children and share this information in their community. The process not only improves the health of mothers and children but it empowers women at the same time. A fourth implementation strategy identified is the provision of community–based services by mobile teams based a peripheral health facilities. These four strategies are not mutually exclusive, of course.

Of the 700 assessments, only four had evidence of mortality impact of 10 years or more, but their common features are striking: they all provided a comprehensive set of primary health care services, including family planning; they had a strong community health worker program that maintained regular contact with all households; they all had strong collaborations with the communities they serve; and they all had strong referral capabilities and provided first–level hospital care.

### Limitations of the evidence identified

Although the evidence is extensive, it does have important limitations that need to be recognized. First of all, the evidence is largely limited to assessments of a small number of interventions implemented over a relatively short period of time (2–3 years) in highly controlled field settings with a relatively small population (only 11% of the projects assessed served more than 25 000 women and children), and almost half (46%) of the projects were implemented over a period of 1 year or less and with only 13% implemented over a period of 5 or more years. Thus, the evidence for effectiveness of more comprehensive programs that reach larger populations over longer periods of time is limited.

There is a notable lack of evidence regarding failed attempts to improve MNCH through CBPHC. Publication bias needs to be recognized, and the overall findings interpreted accordingly. But more importantly, more analyses are needed of the main barriers that hinder the fuller development of CBPHC to improve MNCH and steps that need to be taken to overcome them. Furthermore, more attention needs to be given to the puzzling question of why, given the overwhelming evidence, more effort has not been given to strengthening and scaling up CBPHC, especially in countries with a high burden of maternal, neonatal and child mortality. Ghana is a case in point, where an effective evidence–based CBPHC approach [29] reached only 8% national coverage over an 8–year period as a result of inadequate financial backing and donor support [30,31].

We make no claim that this is a systematic review of the evidence. We do claim that it is a comprehensive review of the evidence. The presence of an *a priori* design, the inclusion of gray literature, the listing of included articles, the presence of a quality assessment of included reviews and incorporation of this into conclusions of individual articles, and the inclusion of conflict of interest and funding information for the entire review allow the review to meet 7 of the 11 quality AMSTAR criteria for judging the quality of a systematic review [32].

Given the broad scope and heterogeneity of the evidence included, by necessity the review is largely descriptive and does not undertake a quantitative analysis of effect strength of specific interventions or packages of interventions. This limits the power of conclusions that pertain to specific interventions. Nonetheless, the main finding of the review, namely that CBPHC is an effective and essential approach for improving MNCH, is not lost by dwelling on detailed discussions of which specific interventions or which packages of interventions are most important. We know that new interventions will continually be introduced in the future, and epidemiological as well contextual conditions will change over time, so keeping a focus on CBPHC as a strategy for implementing specific interventions, which this review attempts to do, is important.

### Strengths of the review

The review described in this series has some important strengths. First, it is one of the most comprehensive in–depth current reviews on this important topic that is highly relevant for accelerating progress in reducing 6 million deaths of mothers and their offspring each year [8,9], most of which are from readily preventable or treatable conditions. While the effectiveness of many of the interventions described here is well–known, the breadth of interventions known to be effective is less well–known, as are the most common strategies used to implement them. The reviewers included evidence not only from the peer–reviewed literature but also from unpublished project evaluations, books, and reports from the gray literature. The review is composed of 700 assessments. Second, it is one of the most comprehensive reviews currently available, with great efforts taken to extract all available information about how each project included in the review was implemented, how communities were engaged, how interventions were delivered at the community level, and what steps were taken to strengthen the health system.

### Estimates of the number of lives of mothers and their children that could be saved by scaling up CBPHC

**Figure 1:**
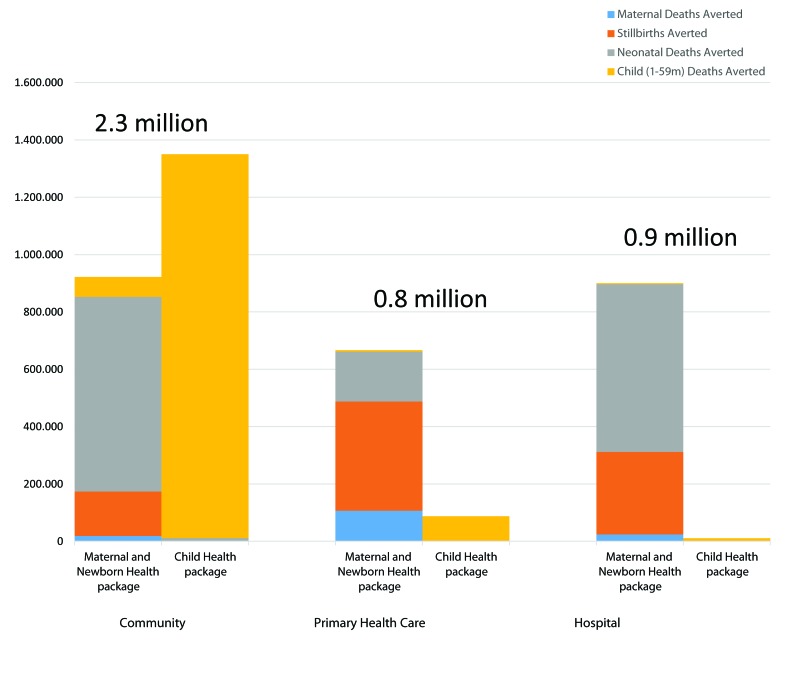
Maternal, perinatal, neonatal and child deaths that can be averted by health–care packages through three service platforms [22]. The numbers above the columns were not in the original figure. The services assumed to be provided in each platform are as follows. ***The community platform:*** all interventions that can be delivered by a community–based health worker with appropriate training and support or by outreach services, such as child health days, immunizations, vitamin A, and other interventions. ***The primary health center (PHC) platform:*** a facility with a doctor or a nurse midwife (or both), nurses and support staff, as well as both diagnostic and treatment capabilities. The PHC provides facility–based contraceptive services, including long–acting reversible contraceptives (implants, intrauterine devices); surgical sterilization (vasectomy, tubal ligation); care during pregnancy and delivery for uncomplicated pregnancies; provision of medical care for adults and children, such as injectable antibiotics, that cannot be done in the community; and training and supervision of community–based workers. ***The hospital platform:*** consisting of both first–level and referral hospitals, includes more advanced services for management of labor and delivery in high–risk women or those with complications, including operative delivery, full supportive care for preterm newborns, and care of children with severe infection or severe acute malnutrition with infection [22].

Long–standing experience and rapidly growing evidence both show that simplified home– and community–based interventions can be remarkably effective in expanding the coverage of evidence–based interventions and reducing maternal, neonatal and child mortality [22,23]. The best current evidence indicates that if the complete package of evidence–based interventions for mothers and their children that can be provided at the community level reach all who need them, 2.3 million deaths would be averted each year compared to the interventions that require delivery in primary health care centers (which would avert 0.8 million deaths) and in hospitals (which would avert 0.9 million deaths) (**Figure 1**) [22].

### Promoting community engagement/empowerment

Promoting community engagement/empowerment to increase intervention effectiveness is obviously not simple, but major progress has been documented [33]. Experience shows that the following questions must be addressed by both programs and communities:

- Will the community be a participating partner and bring its own considerable resources (mostly non–financial) to improve MNCH, or will the more common practices continue of health systems considering communities mainly as targets and essentially passive recipients of services?
- Will the community have the opportunity to participate in setting priorities as well as implementing and evaluating program activities, in contrast to the much more common practice of health professionals defining these roles as the responsibility of the health system?

Although the Expert Panel did not approach these questions as either–or alternatives, it did view community engagement/empowerment as important for enabling the delivery system to more effectively improve MNCH.

Activities that communities can contribute to improving the effectiveness of interventions and that can be empowering for communities include the following:

- Involving local leadership in mobilizing the community for planning and management of activities (including the management of external resources);
- Clarifying local value systems to help both the delivery system and community develop mutual understanding and respect as they work together for results that are effective and equitable;
- Involving women’s groups in participatory learning and action, peer–to–peer education, and provision of home–based care;
- Involving men and mothers–in–law in creative ways that encourage healthy behaviors and appropriate health care utilization;
- Participating in adapting the delivery system to local realities and local culture with integration of interventions for acceptability and efficiency;
- Participating in monitoring, evaluation and accountability; and,
- Collaborating not just in a series of interventions during the initial stages of implementation but establishing long–term partnerships for robust and sustainable systems.

Effective program planning, implementation and assessment require community involvement, and the evidence is clearest for home–based neonatal care and community–based management of childhood diarrhea, pneumonia and malaria. For other interventions (eg, immunizations), community engagement/empowerment is important to ensure that children who need an intervention are taken to where they can receive it (or to take the intervention to where the child is, ie, in the home). CBPHC requires linkages with facilities. Populations with the most limited access to formal health care are typically in the most unreached areas where mortality is the highest and therefore where impact can be greatest. Here also equity issues are central. The nature of effective partnerships between health intervention delivery systems and communities vary greatly as a result of the need to adapt them to the local context [34]. Supportive environments for CBPHC and community engagement/empowerment at local, national, international and global levels are now needed, especially as the evidence of effectiveness continues to grow. Community–level workers providing CBPHC have been shown to be effective in improving not only MNCH in low–income countries but also in improving health priorities in middle– and upper–income countries as well [35,36].

### Scaling up community–based primary health care

The evidence for the effectiveness of CBPHC in improving MNCH at scale is still limited. Yet, encouraging national examples of improvement in MNCH exist in countries such as Afghanistan, Brazil, Ethiopia, Nepal and Rwanda [37,38] and these countries have established strong CBPHC programs that have made a major contribution to these achievements. More research is needed to fully assess the contribution that strengthened CBPHC has made to these achievements.

Innovative approaches to scaling up CBPHC approaches that improve MNCH are needed. Some examples are the following:

- Establishment of a cadre of government–authorized community–level workers throughout the country with gradual addition of responsibilities, as has happened in Afghanistan, Brazil, Bangladesh, Ethiopia, India, Malawi, Nepal, Niger, Rwanda, and many other countries [38];
- The gradual expansion of a package of interventions to national level beginning with a small effective program implemented by one NGO, replication by other NGOs, with gradual transfer of the intervention into the government system as is currently underway in India, as has occurred for home–based neonatal care, beginning with SEARCH’s pioneering work in Gadchiroli [39];
- “Scaling down to scale up” in which a documented successful approach is replicated at other sites with strong local input and flexibility, allowing local champions to emerge, as has been carried out by the Navrongo Initiative working through the Ministry of Health in Ghana [29];
- A three–way partnership at the outset for scaling up, in which the community, government officials, and an outside agent (such as an NGO or technical support group) first establishes model program sites as nodes to adapt and systematize extension to larger populations, as was done in China with the Model Counties Project [40] (which has now become China’s rural MCH system) and as Future Generations has done with its SEED–SCALE approach to improve the health of children in Arunachal Pradesh (India); Tibet (China), Afghanistan and Peru [41];
- A “bottom–up” educational approach to scaling up, in which grassroots workers from many geographic areas and programs in different countries come to a central training center to learn empowerment and CBPHC, as is occurring at the Comprehensive Rural Health Program (CRHP) in Jamkhed, India, where more than 30 000 people from around India and more than 3000 people from 100 other countries have now been trained [42];
- Creation of a national framework giving local communities the option of establishing shared control over health centers and local programs, as has occurred in Peru’s program of *Communidades Locales para la Administracion de Salud* (CLAS), under which one–third of the government’s 2400 health centers are now governed [43]; and,
- The gradual expansion of one key intervention to a national level under the direction of a single NGO, as was carried out by BRAC through its home–based training of mothers to prevent and treat childhood diarrhea [44].

There is a need to test different approaches for rapid scaling up so that CBPHC programs can achieve national impact more rapidly. Even though “command and control” approaches can be used for scaling up standardized components of community–based interventions, in most poor countries such approaches have been supported by external donors for only a limited time period, producing initial successes that cannot be sustained after external funding ends. By contrast, new systematic processes need to be developed that can adapt to local realities in ways that promote community engagement/empowerment and long–term local sustainability [25]. Different approaches to scaling up should be tested through monitoring of quality and coverage as well as through rigorous implementation research. This would enhance the potential for greater effectiveness and long–term sustainability without over–dependence on central or international funding.

The limited evidence of effectiveness of a broad package of CBPHC interventions over a period of more than 3 years at scale is a serious concern. Long–term field studies to assess the ongoing effectiveness of a comprehensive package of CBPHC interventions are needed to enable such programs to continually improve their effectiveness and to provide guidance for similar programs. The strengthening and scaling up of effective CBPHC programs is a long–term process that will require continuing adjustment as conditions and contexts change, and as new evidence-based interventions become available. Efficiencies and final aspects of CBPHC are not adequately address in the literature. Thus, investments in long–term implementation research are greatly needed.

### Specific recommendations of the Expert Panel

The Expert Panel calls for the following steps.

1. CBPHC should be a priority for strengthening health systems, for accelerating progress in achieving universal health coverage, and for ending preventable child and maternal deaths.
2. The amount of resources devoted to CBPHC should be tracked at national and regional levels, and attention should be given by policy makers and political leaders to ensure that funding for CBPHC is expanding appropriately.
3. Communities are an undervalued resource, and their full participation and partnership needs to be fostered in order for CBPHC to reach its full potential. Building partnerships between health systems and communities is essential in order to reach those most in need with effective, equitable, and sustainable programs.
4. Prioritization should be given to strengthening CBPHC in populations with the highest mortality in order to achieve greater impact.
5. A strong CBPHC service delivery platform should be established not only for accelerating progress in improving MNCH and child development but also for reducing the unmet need for family planning, for ending the HIV/AIDS epidemic, controlling malaria, tuberculosis, and priority non–communicable diseases such as hypertension, diabetes and mental illness, and for surveillance (identification of infectious disease outbreaks and registration of vital events). The establishment of the CBHC service delivery platform for MNCH is urgent, while the inclusion of other elements will need to be a gradual and longer–term process. A strong CBPHC service delivery system will make it possible to incorporate new interventions as they are developed, and such a system will be needed for the long term, even after ending preventable child and maternal deaths and achieving universal coverage of health services. Such a system will be needed, in fact, for eventually reaching universal comprehensive health coverage and Health for All.
6. Future progress in improving the effectiveness of CBPHC for MNCH will require an expanded research agenda to continually advance the contextualized evidence on CBPHC program effectiveness at scale over a longer period of time with multiple evidence–based interventions. Adequate financial support for advancing the evidence base for CBPHC program effectiveness will be essential if CBPHC programs are to fulfill their potential.

**Table 2** and **Table 3** provide additional detailed to the recommendations of the Expert Panel for promoting community engagement/empowerment and for strengthening health systems that will make it possible for CBPHC to more effectively reduce maternal, neonatal and child mortality.

**Table 2:** Expert Panel recommendations for promoting community engagement/empowerment for improved maternal, neonatal and child health

Main recommendations	Details
Empower communities and women in these communities to be more actively engaged in improving the health of mothers, newborns and children	Establish a foundation of values that supports partnerships with communities and processes to build community capacity through giving communities a voice in supervising or controlling certain aspects of local government health services, and through building the agency of women (such as the promotion of women’s empowerment, support of micro–credit programs and development of conditional cash transfer programs).
	Support the development of community–based organizations focused on local health needs and on the planning, implementation, and evaluation of local health programs.
Build stronger partnerships between the community and the health system	Create a health system culture that is respectful of and collaborative with community members.
	Create bi–directional communication flows.
	Create bi–directional linkages between the district health system and communities that can help everyone be accountable for health system performance.
Involve communities in monitoring, evaluation, and use of health–related information	Create systems for the community’s generation and use of health data (including registration of births and deaths and identification of those in greatest need of services, as part of a continuing process to promote equity in all stages of health care).
	Develop participatory approaches to the monitoring and evaluation of CBPHC programs, including assessments of mortality impact.

**Table 3:** Expert Panel recommendations for strengthening the delivery system for improved maternal, neonatal and child health

Main recommendations	Details
Extend the delivery system to every community and household	Involve community members in the delivery of services.
	Train and support community–level workers who (1) receive sufficient incentives or salary to support their long–term involvement, (2) receive appropriate supportive and technical supervision from staff based at the nearest health facility, and (3) are accountable to their local community.
	Provide appropriate training and supervision of community–level workers (who preferably are selected from and by the communities where they will work) to perform health tasks that respond to local health needs and that address the epidemiological priorities of mothers and their children.
	Train and support neighborhood volunteers for peer–to–peer health promotion.
	Develop an appropriate balance of community–level workers for the required service intensity (while at the same time ensuring a suitable workload for an appropriate number of tasks and ensuring enough time required for each task, given the distance to homes and the level of remuneration/ incentives).
	Coordinate the activities of the formal health sector with the informal health sector (drug sellers and individual practitioners, including traditional healers).
Promote delivery of interventions to those at greatest risk	Provide “safety nets” that reduce barriers to accessing and providing services (eg, “CBPHC–friendly” insurance systems to remunerate providers and incentive schemes to promote utilization of health services).
	Create equitable service delivery strategies that identify and reach those in greatest need
Build a stronger, more efficient, and more effective health delivery system	Provide adequate, sustainable and flexible global, national and local financing that responds to the needs of community–based programs in relation to the amount being spent for facility–based care.
	Foster investments at the community and local level for support of community–based programs and for strengthening primary health care at peripheral health facilities.
	Provide adequate supplies for service delivery.
	Integrate services at the community level (based on delivery system capacity and local need).
	Monitor expenditures for CBPHC against those for primary health centers and hospitals and ensure that these levels are appropriate given the importance of CBPHC for averting deaths.

Reaching the unreached and most vulnerable members of our global family – namely mothers and children – through CBPHC was the vision of the three global health pioneers – Carl Taylor (founder of the Department of International Health at Johns Hopkins and Chair of the Expert Panel prior to his death in 2010), Jim Grant (Executive Director of UNICEF from 1980 to 1995) and Halfdan Mahler (Director General of WHO from 1973–1988). They all provided leadership for the International Conference on Primary Health Care at Alma–Ata in 1978 and its Declaration of Alma–Ata and worked tirelessly to achieve that vision, which remains unfilled. They recognized, and the Declaration of Alma–Ata affirms, that health care needs to be brought “as close as possible to where people live and work” and that this requires health workers at all levels, including *“physicians, nurses, midwives, auxiliaries and community workers as applicable”* [14]. Over the past three decades, the evidence of what can be achieved through CBPHC to improve the health of mothers, neonates and children has grown exponentially.

However, CBPHC still remains, as El–Saharty and colleagues rightly calls it, an “unfunded afterthought” [45] (p. 270) rather than the solid foundation of effective health systems. Jim Grant repeatedly reminded us that “morality must march with changing capacity” [46]. And Halfdan Mahler reminded the world in his 2008 address to the 61st World Health Assembly, “unless we all become partisans in the renewed local and global battles for social and economic equity in the spirit of distributive justice, we shall indeed betray the future of our children and grandchildren” [47]. Establishing the political will to fund and build strong CBPHC programs is urgently needed, as is defining the resource needs so that these programs will not remain an “unfunded afterthought.”

Carl Taylor, in his final publication, wrote that “[r]eal social change occurs when officials and people with relevant knowledge and resources come together with communities in joint action around mutual priorities” [34]. The evidence confirms the promise of CBPHC in ending preventable maternal, neonatal and child deaths. Building on this evidence and making CBPHC the priority that it needs to be is one of the great challenges for global health in the 21st century and one of the giant steps that can be taken to eventually achieve Health for All.

## CONCLUSIONS

Stronger CBPHC programs that foster community engagement/empowerment and implement evidence–based interventions will be essential for achieving universal coverage of health services by 2030 (as called for by the Sustainable Development Goals recently adopted by the United Nations) [48]), ending preventable child and maternal deaths by 2030 (as called for by the World Health Organization, UNICEF and many other countries) [17], and eventually achieving Health for All as initially envisioned in 1978 at the International Conference on Primary Health Care convened by WHO and UNICEF [14]. Stronger CBPHC programs will create entry points and synergies for expanding the coverage of family planning services [49] and for accelerating progress in the detection and treatment of HIV/AIDS [50], tuberculosis [51] malaria [52], and hypertension and other chronic diseases [53]. International cooperation will be important in promoting stronger CBPHC implementation world–wide. Advocacy at global, international, national and local levels, exchange of information and experiences, training, and evaluations of program implementation will all contribute to stronger CBPHC programming. Specific mechanisms need to be developed through which we can more effectively learn from experience and generate evidence to guide local, national and international policies and programs.
