# Supplementary material for: Comprehensive review of the evidence regarding the effectiveness of community–based primary health care in improving maternal, neonatal and child health: 8. summary and recommendations of the Expert Panel
Source: J Glob Health. 2017 Jun 29;7(1):010908. doi: 10.7189/jogh.07.010908 (PMC5475312; doi:10.7189/jogh.07.010908)
Supplement: Online Supplementary Document [file jogh-07-010908-s001.pdf]

Online Supplementary Document

Black et al. Comprehensive review of the evidence regarding the effectiveness of COMMUNITY–BASED PRIMARY HEALTH CARE in improving maternal, neonatal and child health: 8. summary and recommendations of the Expert Panel

J Glob Health 2017;7:010908

Expert Panel Members, Task Force Members, and Workshop Participants

Over the period of the review, the composition of the Expert Panel changed somewhat. A full description of the persons who participated in the Panel at each stage of the Panel activity is provided elsewhere [1]. Here we list those who attended the 2008 meeting in New York City and those who participated in the preparation/review of this article.

Expert Panel Members who participated in the 2008 meeting and/or review of this article

Name	Participated in 2008 meeting in New York City	Designation at time of 2008 meeting (if present)	Current designation (if it has changed since 2008)	Participated in the review and finalization of this article
Shobha Arole			Jamkhed Comprehensive Rural Health Project	X
Abhay Bang	X	Society for Education, Action and Research (SEARCH), Gadchiroli, India		X
Zulfiqar Ahmed Bhutta			Centre for Global Child Health, Hospital for Sick Children, Toronto, Canada and Center of Excellence in Women and Child Health, the Aga Khan University, Karachi, Pakistan	X
Robert Black	X	Department of International Health, Johns Hopkins University, Baltimore, Maryland, USA		X
Mushtaque Chowdhury			BRAC, Dhaka, Bangladesh	X
Anthony Costello			World Health Organization, Geneva, Switzerland	
Dan Kasaje	X	Tropical Institute of Community Health and Development, Kisumu, Kenya		
Betty Kirkwood			London School of Hygiene and Tropical Medicine, London, UK	X

Name	Participated in 2008 meeting in New York City	Designation at time of 2008 meeting (if present)	Current designation (if it has changed since 2008)	Participated in the review and finalization of this article
Rudolph Knippenberg			Senior Advisor for Health. UNICEF, New York City (currently retired)	
Nazo Kureshy	X	Child Survival and Health Grants Program, Bureau of Global Health, USAID, Washington, DC		X
Claudio Lanata	X	Institute of Nutritional Research, Lima, Peru		X
Adetokunbo Lucas	X	Department of International Health, Harvard University School of Public Health, Boston, MA, USA		
James Phillips	X	Mailman School of Public Health, Columbia University, New York City, NY, USA		X
Pang Ruyan		World Health Organization, Beijing, China		
David Sanders	X	School of Public Health, University of Western Cape, Cape Town, South Africa		
Agnes Soucat			World Health Organization, Geneva, Switzerland	
Carl Taylor (now deceased)	X	Department of International Health, Johns Hopkins University, Baltimore, Maryland, USA, and Future Generations, Franklin, WV, USA		
Mary Taylor	X	The Bill and Melinda Gates Foundation, Seattle, WA, USA	Independent consultant, South Royalton, Vermont, USA	X
Cesar Victora			Federal University of Pelotas, Pelotas, Brazil	X
Zonghan Zhu	X	Capital Institute of Pediatrics and China Advisory Center for Child Health, Beijing, China		X

Other participants in the 2008 UNICEF meeting in New York City included: Ann Veneman, Executive Director, UNICEF; Elizabeth Mason, Director, Child and Adolescent, WHO; Sadia Chowdhury, Senior Advisor, the World Bank; Nicholas Alipui, Director, Programme Division, UNICEF; Clarissa Brocklehurst, Associate Director for Water and Environmental Sanitation,

UNICEF; Jimmy Kolker, Associate Director for HIV/AIDS, UNICEF; Peter Salama, Associate Director for Health, UNICEF; Werner Schultink, Associate Director for Nutrition, UNICEF.

Members of the Writing Group included the following: Robert Black, Carl Taylor (now deceased), and Henry Perry.
